# S35. Investigating the ICOS/ICOSL pathway as a target for combination therapy with anti-CTLA-4

**DOI:** 10.1186/2051-1426-2-S2-I6

**Published:** 2014-03-12

**Authors:** P Sharma

**Affiliations:** 1 MD Anderson Cancer Center, Department of Genitourinary Medical Oncology Unit Number: Unit 18-7, Houston TX, USA

## 

Biomarker studies used with immunotherapeutic strategies in the clinic have typically involved monitoring immunologic changes within the systemic circulation; however, recent data indicate that immunological changes within tumor tissues will be more likely to predict clinical responses. To obtain such data we conducted the first pre-surgical clinical trial with anti-CTLA-4 (ipilimumab) in a cohort of patients with localized bladder cancer. We are also conducting the first combination therapy pre-surgical trial with ipilimumab plus leuprolide acetate in patients with localized prostate cancer. Immunological data from these trials are obtained from both tumor tissues and blood samples.

We found an increased frequency of CD4 and CD8 T cells expressing high levels of inducible costimulator (ICOS) and decreased frequency of FOXP3-expressing CD4 T cells within tumor tissues of treated patients. The CD4^+^ICOS^hi^ population contained effector T cells that produced IFN-g and recognized the cancer-testis antigen NY-ESO-1 expressed on tumor cells. We therefore identified ICOS as marker of a subset of effector T cells that is increased in frequency after anti-CTLA-4 therapy. ICOS^+^ T cells are being explored as both a pharmacodynamic marker for treatment with anti-CTLA-4 as well as a novel target to improve the efficacy of anti-CTLA-4 therapy.

These observations led us to test the possibility that engagement of ICOS could enhance the efficacy of anti-CTLA-4 therapy. To this end we transduced mouse B16F10 melanoma cells with a cDNA encoding ICOSL or a control construct. B16-ICOSL+ cells (IVAX) and control B16 cells were irradiated and used alone or in combination with anti-CTLA-4 to treat mice bearing established B16F10 tumors. We found that combination of the IVAX with anti-CTLA-4 was markedly more effective than the control vaccine plus anti-CTLA-4 or that of any single treatment alone. The increase in therapeutic efficacy was accompanied by a marked in increase in the density and functionality of CD4 and CD8 T cells within the tumor.

These results suggest a novel strategy for manipulating the immune system to enhance anti-tumor responses: checkpoint blockade coupled with provision of agonist signals to enhance costimulation mediated by ICOS.

